# A Robust Nonlinear Observer for a Class of Neural Mass Models

**DOI:** 10.1155/2014/215943

**Published:** 2014-03-20

**Authors:** Xian Liu, Dongkai Miao, Qing Gao

**Affiliations:** Key Lab of Industrial Computer Control Engineering of Hebei Province, Institute of Electrical Engineering, Yanshan University, Qinhuangdao 066004, China

## Abstract

A new method of designing a robust nonlinear observer is presented for a class of neural mass models by using the Lur'e system theory and the projection lemma. The observer is robust towards input uncertainty and measurement noise. It is applied to estimate the unmeasured membrane potential of neural populations from the electroencephalogram (EEG) produced by the neural mass models. An illustrative example shows the effectiveness of the proposed method.

## 1. Introduction

Mathematical modelling provides a powerful tool for studying mechanisms involved in the generation of different electroencephalogram (EEG) rhythms and neuronal processes of neurological disorders. There are two types of approaches to model neural signals. One is on the basis of networks built with a large number of elementary cells to describe the activity of a given system. The other is a lumped-parameter approach in which neural populations are modeled as nonlinear oscillators. Neural mass models are based on the latter approach. These models comprise macrocolumns or cortical areas and represent the mean activity of the whole population by using one or two state variables. It is seldom tractable to model EEG signals at the neuronal level due to the complexity of real neural networks. The use of neural mass models has been the preferred approach since 1970s. Neural mass models originated from the seminal work of Lopes da Silva et al. for alpha rhythm generation [1] and redesigned by Jansen and Rit to represent the generation of evoked potentials in the visual cortex [2]. The dynamical analysis [3–6] and control [7–9] of the neural mass models have been widely studied over the years. Despite the existence of these neural mass models for simulating distinct rhythms in EEG signals, neural activity is always measured through observing just a single variable such as voltage. A combination of noise in neurons and amplifiers as well as uncertainties in recording equipment leads to uncertainty of the measurement. The observation of states therefore plays significant roles in neuroscientific studies for better understanding of the human brain [10].

In general, neural mass models can be expressed as nonlinear systems of Lur'e type [11]. Observer design for nonlinear systems of Lur'e type [12–14] and the neural mass models [15] has been widely investigated over the years. We here introduce a new method of designing a robust nonlinear observer for the neural mass models. The Lur'e system theory and new tools in linear matrix inequality (LMI) method [16] are used to obtain the new reformulation. We should mention that this new reformulation takes input uncertainty and measurement noise into account. The superiority of the proposed method is demonstrated in the last section which is devoted to numerical comparisons.

*Notation*. The identity matrix is denoted by *I*. The symmetric block component of a symmetric matrix is denoted by ⋆. The vector norm is denoted by |·|. The *L*_2_ norm is denoted by ||·||_2_. The set of positive real numbers is denoted by ℝ_+_.

## 2. Problem Formulation

Let us consider a class of neural mass models that can be formulated as the following mathematical structure:

(1) $$\begin{array}{c} \dot{x}=Ax+Bf\left(Hx\right)+B_{1}u,\\ z=Cx+Dw, \end{array}$$

where *x* ∈ ℝ^*n*^ is the state vector, *u* ∈ ℝ^*p*^ is the input, *z* ∈ ℝ^*q*^ is the measurement output, *w* ∈ ℝ^*s*^ is the measurement noise, *A* ∈ ℝ^*n*×*n*^, *B* ∈ ℝ^*n*×*m*^, *B*_1_ ∈ ℝ^*n*×*p*^, *C* ∈ ℝ^*q*×*n*^, *H* ∈ ℝ^*m*×*n*^, and *D* ∈ ℝ^*q*×*s*^ are constant matrices, and *f*(·): ℝ^*n*^ → ℝ^*m*^ is a memoryless nonlinear vector valued function which is continuously differentiable on ℝ^*m*^. Each entry of the state-dependent nonlinearity *f*(*Hx*) is a function of a linear combination of the states

(2) $$\begin{array}{c} f_{l}=f_{l}\left(\sum_{j=1}^{n}h_{lj}x_{j}\right)=f_{l}\left(h_{l}x\right),\;l=1,2,\ldots ,m, \end{array}$$

where $h_{l}=\left[\begin{array}{ccc} h_{l1} & \cdots & h_{ln} \end{array}\right]$. It satisfies certain slope-restricted condition

(3) $$\begin{array}{c} 0\leq f_{l}^{\prime }\left(\sigma \right)\leq \delta_{l},\;\forall \sigma \in \mathbb{R},\;\;l=1,2,\ldots ,m, \end{array}$$

where *δ*_*l*_ ≥ 0. The models in David and Friston [4], Goodfellow et al. [6], Jansen and Rit [2], and Wendling et al. [3] can all be expressed as the form of (1). Let us construct the following observer for plant (1):

(4) $$\begin{array}{c} \dot{\hat{x}}=A\hat{x}+Bf\left[H\hat{x}+K\left(C\hat{x}-z\right)\right]+L\left(C\hat{x}-z\right)+B_{1}\left(u+d\right), \end{array}$$

where $\hat{x}$ is the estimation of state, *d* ∈ ℝ^*p*^ is the disturbance of input, and *K* ∈ ℝ^*m*×*q*^, *L* ∈ ℝ^*n*×*q*^ are the observer matrices to be designed. Defining the observer error as $e=\hat{x}-x$, the dynamics of it are governed by

(5) $$\begin{array}{c} \dot{e}=\left(A+LC\right)e+B_{1}d-LDw+B\eta \left(e,t\right), \end{array}$$

where *η*(*e*, *t*) = *f*(*V*) − *f*(*U*), $V=H\hat{x}+K(C\hat{x}-z)$, and *U* = *Hx*. Note from (3) that each entry of the nonlinearity *η*(*e*, *t*) satisfies

(6) 0≤ηl(e,t)vl−ul=fl(vl)−fl(ul)vl−ul≤δl, ∀e∈ℝ,           ∀t∈ℝ+,  l=1,2,…,m.

The observer design for (1) consists in finding observer matrices *K* and *L* such that the observer error *e* satisfies the following property for all *t* ≥ 0:

(7) $$\begin{array}{c} {||e||}_{2}\leq \kappa \left|e_{0}\right|+\rho_{d}{||d||}_{2}+\rho_{w}{||w||}_{2}, \end{array}$$

where scalars *κ* > 0, *ρ*_*d*_ ≥ 0, and *ρ*_*w*_ ≥ 0. The disturbance gains from *d* and *w* to *e* are *ρ*_*d*_ and *ρ*_*w*_.

## 3. Main Results

**Theorem 1**

Consider plant (1) and observer (4). Under the slope restrictions (3), if there exist a matrix *P* = *P* ^*T*^ > 0, a diagonal matrix *M* = diag⁡(*m*_1_,…, *m*_*m*_) ≥ 0, matrices *K* and *L*, nonsingular matrices *G* and *F* with appropriate dimensions, and scalar constants *ε* > 0, *μ*_*w*_ ≥ 0, and *μ*_*d*_ ≥ 0 such that

(8) $$\begin{array}{c} \left[\begin{array}{ccccc} G+G^{T} & \Gamma_{1} & GB & GB_{1} & -GLD\\ ⋆ & \Gamma_{2} & \Gamma_{3} & FB_{1} & -FLD\\ ⋆ & ⋆ & -M & 0 & -\frac{1}{2}\Delta MKD\\ ⋆ & ⋆ & ⋆ & -\mu_{d}I & 0\\ ⋆ & ⋆ & ⋆ & ⋆ & -\mu_{w}I \end{array}\right]\leq 0,\\ \Gamma_{1}=G\left(A+LC\right)+P-F^{T},\\ \Gamma_{2}=F\left(A+LC\right)+{\left(A+LC\right)}^{T}F^{T}+\varepsilon I,\\ \Gamma_{3}=FB+\frac{1}{2}{\left(H+KC\right)}^{T}M\Delta , \end{array}$$

then the observer error *e* satisfies (7) for all *t* ≥ 0, where  Δ = diag⁡(*δ*_1_,…, *δ*_*m*_), $\kappa =\sqrt{\lambda_{\max}(P)/\varepsilon }$,$\;\;\rho_{d}=\sqrt{\mu_{d}/\varepsilon },$ and $\rho_{w}=\sqrt{\mu_{w}/\varepsilon }$.

**Proof**

The inequality (8) can be written as

(9) $$\begin{array}{c} \Omega +V_{1}\left[\begin{array}{c} G\\ F \end{array}\right]V_{2}+V_{2}^{T}{\left[\begin{array}{c} G\\ F \end{array}\right]}^{T}V_{1}^{T}\leq 0, \end{array}$$

where

(10) $$\begin{array}{c} \Omega =\left[\begin{array}{ccccc} 0 & P & 0 & 0 & 0\\ ⋆ & \varepsilon I & \frac{1}{2}{\left(H+KC\right)}^{T}M\Delta & 0 & 0\\ ⋆ & ⋆ & -M & 0 & -\frac{1}{2}\Delta MKD\\ ⋆ & ⋆ & ⋆ & -\mu_{d}I & 0\\ ⋆ & ⋆ & ⋆ & ⋆ & -\mu_{w}I \end{array}\right],\\ V_{1}={\left[\begin{array}{ccccc} I & 0 & 0 & 0 & 0\\ 0 & I & 0 & 0 & 0 \end{array}\right]}^{T},\\ V_{2}=\left[\begin{array}{ccccc} -I & A+LC & B & B_{1} & -LD \end{array}\right]. \end{array}$$

By using the well-known projection lemma in LMI method [16], (9) can be transformed into

(11) $$\begin{array}{c} \left[\begin{array}{cccc} \Pi_{1} & \Pi_{2} & PB_{1} & -PLD\\ ⋆ & -M & 0 & -\frac{1}{2}\Delta MKD\\ ⋆ & ⋆ & -\mu_{d}I & 0\\ ⋆ & ⋆ & ⋆ & -\mu_{w}I \end{array}\right]\leq 0, \end{array}$$

where

(12) $$\begin{array}{c} \Pi_{1}={\left(A+LC\right)}^{T}P+P\left(A+LC\right)+\varepsilon I,\\ \Pi_{2}=PB+\frac{1}{2}{\left(H+KC\right)}^{T}M\Delta . \end{array}$$

The derivative of *V*(*e*) = *e* ^*T*^ *Pe* is given by

(13) V˙=e˙TPe+eTPe˙≤e˙TPe+eTPe˙−∑l=1mmlηl(e,t)[ηl(e,t)−δl(vl−ul)].‍  

Applying (11), we have

(14) $$\begin{array}{c} \dot{V}\leq -\varepsilon e^{T}e+\mu_{d}d^{T}d+\mu_{w}w^{T}w \end{array}$$

from which it follows that

(15) $$\begin{array}{c} {||e||}_{2}\leq \sqrt{\frac{\lambda_{\max\left(P\right)}}{\varepsilon }}\left|e_{0}\right|+\sqrt{\frac{\mu_{d}}{\varepsilon }}{||d||}_{2}+\sqrt{\frac{\mu_{w}}{\varepsilon }}{||w||}_{2}. \end{array}$$

Hence, (7) results from $\kappa =\sqrt{\lambda_{\max}(P)/\varepsilon }$,$\;\;\rho_{d}=\sqrt{\mu_{d}/\varepsilon }$, and $\rho_{w}=\sqrt{\mu_{w}/\varepsilon }$.

Theorem 1 shows that the observer design for (1) consists in finding observer matrices *K* and *L* to satisfy (8) with a symmetric matrix *P* > 0, a diagonal matrix *M* ≥ 0, nonsingular matrices *G*, *F*, and scalar constants *ε* > 0, *μ*_*w*_ ≥ 0, and *μ*_*d*_ ≥ 0. The feasible solution of (8) can be obtained by solving the following optimization problem:

(16) min⁡max⁡⁡ {μw,μd}s.t.  (8),  P>0, M≥0, ε>0, μw≥0, μd≥0.

Efficient numerical tools such as YALMIP in MATLAB are available for this task. Once the values of *μ*_*w*_ and *μ*_*d*_ are computed, the disturbance gains *ρ*_*w*_ and *ρ*_*d*_ can also be derived. When no input uncertainty and measurement noise are taken into account, Theorem 1 is simplified as follows.

**Theorem 2**

Consider plant (1) and observer (4) with *d* = 0 and *w* = 0. Under the slope restrictions (3), if there exist a matrix *P* = *P* ^*T*^ > 0, a diagonal matrix *M* = diag⁡(*m*_1_,…, *m*_*m*_) ≥ 0, matrices *K*, *L*, nonsingular matrices *G*, *F*, and scalar constants *ε* > 0, *μ*_*w*_ ≥ 0 such that

(17) $$\begin{array}{c} \left[\begin{array}{ccc} G+G^{T} & \Gamma_{1} & GB\\ ⋆ & \Gamma_{2} & \Gamma_{3}\\ ⋆ & ⋆ & -M \end{array}\right]\leq 0, \end{array}$$

where Γ_1_, Γ_2_, and Γ_3_ are defined as Theorem 1, then the origin of the observer error system (5) is globally exponentially stable.

## 4. Simulations

Let us consider a neural mass model developed by Jansen and Rit [2]. This type of single cortical column model with altered parameters is able to generate realistic patterns such as alpha rhythms and epileptiform spikes in EEG. It can be formulated as the form of (1) with the state vector *x* = [*x*_1_ *x*_2_ *x*_3_ *x*_4_ *x*_5_ *x*_6_ *x*_7_ *x*_8_]^*T*^, where *x*_*i*_  (*i* = 1,3, 5,7) are the mean membrane postsynaptic potentials and *x*_*j*_  (*j* = 2,4, 6,8) are their time derivatives. The input *u* is the afferent influence from neighbouring or more distant columns and is modeled by a Gaussian white noise with mean value 90 and standard deviation 30. The output *z* is the EEG measurement available to the observer. The system matrices are as follows:

(18) A=diag⁡(A1,…,A4),Ai=[01−κi2−2κi], κ1=κ2=a, κ3=b,       κ4=ad,B1=[000θaa0000]T,C=[0010−1000],  D=1,B=[0θaa00000θaad000θaaC2000000000θbbC400]T,H=[0010−1000C10000000C30000000],f(Hx)=[S(x3−x5)S(C1x1)S(C3x1)]T,S(v)=2e01+er(v0−v).

The function *S*(·) satisfies (3) with *δ*_*l*_ = (1/2)*e*_0_ *r*  (*l* = 1,2, 3). All values of the constants in the model are set on a physiological interpretation basis which can be found in [2]. The standard values of these constants are given anatomically as

(19) $$\begin{array}{c} \theta_{a}=3.25\;\text{mV},\;\;\theta_{b}=22\;\text{mV},\;a=100\;{\text{s}}^{-1},\;\;b=50\;{\text{s}}^{-1},\\ v_{0}=6\;\text{mV},\;\;e_{0}=2.5\;{\text{s}}^{-1},\\ r=0.56\;{\text{mV}}^{-1},\;\;a_{d}=33\;{\text{s}}^{-1},\;\;C_{1}=135,\\ C_{2}=108,\;\;C_{3}=33.75,\;\;C_{4}=33.75. \end{array}$$

**Figure 1:**
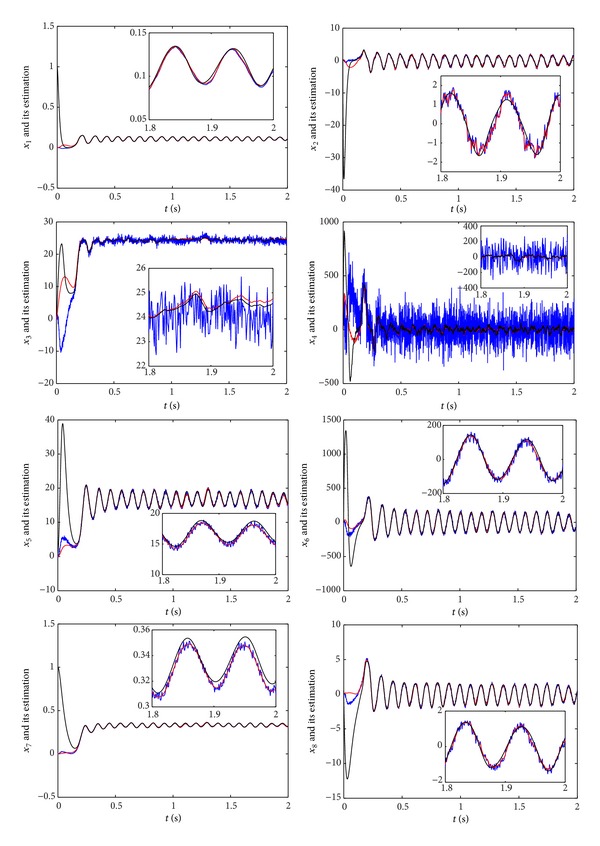
The time evolutions of the states *x*_1_–*x*_8_ and their estimations.

We design the robust nonlinear observer (4) for the neural mass model. The performance of the observer obtained from Theorem 1 is presented in what follows. Input disturbance *d* ~ *N*(0,0.1^2^) and measurement noise *w* ~ *N*(0,0.9^2^) are introduced in the design of robust nonlinear observer. For the robust nonlinear observer, we solve the optimization problem (16) to obtain *K* and *L*. The computed disturbance gains *ρ*_*w*_ = 596 and *ρ*_*d*_ = 3.53 are derived by using the YALMIP toolbox in MATLAB. They are much less than the values given in [15]. In the following simulations, the initial states of the neural mass model and the observer are chosen as *x*(0) = [1  0.5  1  0.5 1  0.5  1  0.5]^*T*^ and $\hat{x}\left(0\right)={\left[0\;\;0\;\;0\;\;0\;\;0\;\;0\;\;0\;\;0\right]}^{T}$, respectively.  Figure 1 presents the time evolutions of the states *x*_1_–*x*_8_ (black lines) and their estimations, that is, the states of observer (4) proposed in this study (red lines) and in [15] (blue line). Insets are given to show the zoom-in on data.  Figure 1 shows that the states of observer (4) obtained from Theorem 1 do converge to a neighbourhood of the states of the neural mass model. It also shows that the observer proposed in this study performs better than that proposed in [15].

## 5. Conclusions

We have designed a robust nonlinear observer for a class of neural mass models by using the Lur'e system theory and the projection lemma. The resulting observer inhibits input uncertainty and measurement noise. We apply this observer to the neural mass model that generates alpha rhythms to estimate the mean membrane potential of neural populations from the EEG measurement. We show that the proposed observer performs better than some existing ones. The proposed method can also be applied to other types of neural models that have the typical structure of Lur'e systems.
